# Gluten-degrading bacteria: availability and applications

**DOI:** 10.1007/s00253-021-11263-5

**Published:** 2021-04-10

**Authors:** Viia Kõiv, Tanel Tenson

**Affiliations:** Institute of Technology, University of Tartu, Tartu, Estonia

**Keywords:** Celiac disease, Peptidases, Food, Environment

## Abstract

**Abstract:**

Gluten is a mixture of storage proteins in wheat and occurs in smaller amounts in other cereal grains. It provides favorable structure to bakery products but unfortunately causes disease conditions with increasing prevalence. In the human gastrointestinal tract, gluten is cleaved into proline and gluten rich peptides that are not degraded further. These peptides trigger immune responses that might lead to celiac disease, wheat allergy, and non-celiac gluten sensitivity. The main treatment option is a gluten-free diet. Alternatively, using enzymes or microorganisms with gluten-degrading properties might alleviate the disease. These components can be used during food production or could be introduced into the digestive tract as food supplements. In addition, natural food from the environment is known to enrich the microbial communities in gut and natural environmental microbial communities have high potential to degrade gluten. It remains to be investigated if food and environment-induced changes in the gut microbiome could contribute to the triggering of gluten-related diseases.

**Key points:**

• *Wheat proteins, gluten, are incompletely digested in human digestive tract leading to gluten intolerance.*

• *The only efficient treatment of gluten intolerance is life-long gluten-free diet.*

• *Environmental bacteria acquired together with food could be source of gluten-degrading bacteria detoxifying undigested gluten peptides.*

## Introduction

Gluten is a mixture of grain storage proteins in cereal grains. The major gluten source in the human diet is wheat, domesticated approximately 10,000 years ago when hunter-gatherers settled in the Middle East. Since then, wheat has been an integral part of the Western type of diet. The dough made from wheat flour has unique baking qualities which provides it with advantages over other crops in temperate regions. The properties of wheat dough depend on the major grain endosperm storage proteins, which together form the “gluten” protein fraction. The major components of gluten are responsible for providing “viscoelasticity” while making leavened bread, but also unleavened bread, cookies, pasta, and many other food products. As a consequence, the total protein concentration in the grain is the most widely applied criteria for wheat baking quality, and an important factor in the price of wheat (Barneix 2007).

Despite 10,000 years of coexistence, gluten-rich food is new for humans—we lack the enzymes that can fully degrade gluten proteins. As a consequence, consumption of wheat products may trigger an autoimmune enteropathy, celiac disease (CD), in genetically susceptible individuals. The genetic predisposition depends on the human leukocyte antigen (HLA) type, specifically the DQ2 or DQ8 alleles (Van Heel et al. 2007; Dubois and Van Heel 2008). However, it has been shown that only 3% of genetically susceptible persons develop the disease; therefore, possessing a predisposing HLA risk allele is necessary but not sufficient to develop CD (Pozo-Rubio et al. 2012; Chibbar and Dieleman 2019). A constant increase in the prevalence of CD has been well documented, and while some of this increase can be attributed to improvements in the complex diagnostic procedures (Kelly et al. 2015), it is also evident that the prevalence of CD is growing worldwide. This can be related to changes in our diet and environmental factors: the increased quantity of ingested gluten, infant feeding patterns, the spectrum of intestinal infections, and gut microbiota colonization (Mustalahti et al. 2010; Dydensborg et al. 2012; Chan et al. 2017; Dydensborg Sander et al. 2019). Moreover, new diseases related to wheat consumption have emerged such as wheat allergy (WA) and non-celiac gluten sensitivity (NCGS) (Inomata 2009; Sapone et al. 2011; Pietzak 2012). Together with CD, these disorders are referred to as gluten intolerance.

The only effective way for CD treatment is a life-long gluten-free diet. The products that have been specially processed to reduce the gluten content can be labeled “gluten-free” if the total gluten content is below 20 mg/kg. Maintaining a gluten free diet is problematic, because it is often not commercially accessible to everyone (Newberry 2019). In addition, small amounts of the gluten components can still be present in food due to either inefficient processing or cross-contamination. Therefore, alternative strategies are needed.

Recent research has shown that gluten and gluten-derived peptides can be degraded by peptidases from different sources. These peptidases can either be used to produce gluten-free foods from gluten-containing raw materials (Rizzello et al. 2007), or they have been suggested as an oral therapy for CD, in which dietary gluten is hydrolyzed by coingested peptidases already in the mouth or stomach, thus preventing CD-specific immune reactions in the small intestine. Oral enzyme therapy is considered a promising candidate to assist gluten-free diet (Plugis and Khosla 2015). Glutenases used for this purpose originate from environmental species of bacteria, fungi, plants, and insects.

A promising approach involves relying on living microorganisms to alleviate the gluten-triggered conditions. Here, we review potential microbes that could be used for this purpose and discuss the sources that could be relevant for enrichment of food with gluten-degrading microbes.

## Gluten

Wheat is currently the most important crop in the world and together with maize and rice accounts for over 70% of the total production of cereals. About 95% of the wheat grown worldwide is hexaploid, with most of the remaining 5% being tetraploid durum wheat, also termed pasta wheat, which is mostly cultivated in the dry Mediterranean region. Hexaploid bread wheat (*Triticum aestivum* L. ssp. *aestivum*) derives its three genomes (A, B, and D) from three diploid wild ancestors: *Triticum urartu* (AA), an unknown close relative of *Aegilops speltoides* (BB), and *Ae. tauschii* (DD) (Marcussen et al. 2014). The initial allopolyploidization event is hypothesized to have involved the A and B genome donors, giving rise to tetraploid emmer wheat (*T. turgidum*; AABB). This species subsequently hybridized with the D genome donor *Ae. tauschii* to form modern hexaploid bread wheat (AABBDD). Durum wheat *Triticum turgidum* L. spp. *dicoccum*, genome AABB is derived from the ancient *T. turgidum* (AABB) (Mefleh et al. 2019). Therefore, the wild emmer wheat is the progenitor of both cultivated hexaploid bread wheat and tetraploid pasta wheat.

Although the protein content in cereal grains is relatively low (about 10–20% of dry weight), in modern wheat, 80–90% of the total protein is gluten (Bromilow et al. 2017). The precise number of different gluten proteins present in mature seeds can vary, but is probably between 50 and 100. Traditionally, gluten proteins have been classified into roughly equal fractions according to their solubility in alcohol–water solutions (e.g., 60% ethanol): the soluble gliadins and the insoluble glutenins (Obsborn 1924). However, based on their composition, both gliadins and glutenins can be referred to as prolamins. The gliadins comprise monomeric subunits which are further classified into α-, γ-, and ω-gliadin fractions. The glutenins comprise two groups of subunits, high molecular weight (HMW) and low molecular weight (LMW) glutenin subunits, which form alcohol-insoluble polymers stabilized by inter-chain disulfide bonds. However, amino acid sequences show that gliadins and glutenin subunits are related. Therefore, a new classification divides all prolamins into three groups in relation to structural and evolutionary relationships: sulfur-rich (S-rich); sulfur-poor (S-poor); and high molecular weight (HMW) prolamins (Shewry and Tatham 1990; Shewry and Halford 2002) (Table 1.). A characteristic of all prolamins is the presence of large domains of short repetitive peptide sequence motifs dominated by proline and glutamine residues. An important feature is the presence of cysteine residues that link prolamin proteins through intra- or intermolecular disulfide bonds, forming a continuous network in dough.

**Table 1:** Classification of gluten prolamins from wheat, barley, rye, and oat, based on Shewry and Halford (2002), Wieser and KoeHler (2012), and Shewry (2019)

Type of gluten prolamins	Partial amino acid composition (mol %)	Components	State	Repetitive unit	Proportion of prolamin fraction (%)	Grain species
HMW prolamins	30–35% glutamine, 10–16% proline, 15–20% glycine, 0.5–1.5% cysteine, 0.7–1.4% lysine	HMW subunit of glutenin	Polymeric	QQPGQG; GYYPTSPQQ	6–10%	Wheat
		D-hordein	Polymeric	QQPGQG	2–4%	Barley
		HMW secalin	Polymeric	QQPGQG	2%	Rye
S-rich prolamins	30–40% glutamine, 15–20% proline, 2–3% cysteine, <1% lysine	γ-gliadin	Monomeric	PQQPFPQ	70–80%	Wheat
		α-gliadin	Monomeric	QPQPFP; PQQPY		
		B- and C-type LMW subunit of glutenin	Polymeric	QPQQPFP		
		B-hordein	Monomeric	QQPFPQ	80%	Barley
		γ-hordein	Polymeric	QPQQPFP		
		γ-secalins	Poly/Monomeric	(Q)QPQQPFP	80%	Rye
S-poor prolamins	40–50% glutamine, 20–30% proline, 0–0.5% lysine, 0 cysteine, 1 cysteine residue in D-type LMW subunit	ω-gliadin	Monomeric	PQQPFPQQ	10–20%	Wheat
		D-type LMW subunits of glutenin	Polymeric	PQQPQQ		
		C-hordein	Monomeric	PQQPFPQQ	10–15%	Barley
		ω-secalin	Monomeric	QPQQPFP	10–15%	Rye
Other gluten prolamins	23–29% glutamine, 8–10% proline, 6.5–8.5% valine, 3.5–5% cysteine	Avenin	Monomeric	PFVQQQQ	a	Oat

*S-rich*, sulfur-rich; *S-poor*, sulfur-poor; *HMW*, high molecular weight; *LMW*, low molecular weight; *a*, 10–20% of total proteins (Anderson 2014)

In addition to wheat, a prolamin fraction also exists in related cereal grains that belong to the family *Poaceae* such as rye (*Secale cereale*) (secalin), barley (*Hordeum vulgare*) (hordein), and oat (*Avena sativa*) (avenin) albeit in much lower quantities. The sequences within these proteins are similar, but unique bread making properties are provided only by wheat gluten and to a limited extent rye gluten.

### Gluten-related disorders

The extensive proline- and glutamine-rich repeated sequences of wheat seed storage proteins are responsible for bread quality, yet unfortunately play a crucial role in triggering hypersensitivity reactions such as celiac disease (CD), wheat allergy (WA), and non-celiac gluten sensitivity (NCGS) (Scherf et al. 2016).

### Celiac disease

Celiac disease (CD) is an autoimmune enteropathy caused by genetic and environmental factors with an estimated worldwide prevalence of about 1% of the global population (Mustalahti et al. 2010). The symptoms are malabsorption, steatorrhea, weight loss, or growth failure (Kelly et al. 2015). Ingested food proteins are usually digested into amino acids, dipeptides, and tripeptides by gastric, pancreatic, and brush border proteases. Proline-rich sequences of prolamines are partially resistant to enzymatic degradation in the gastrointestinal tract that results in relatively long peptide fragments. These undigested proline- and glutamine repetitive peptide fragments pass through the epithelial barrier of the small intestine and reach the lamina propria where transglutaminase (tTG) deaminates the selected glutamine residues, thereby enhancing their affinity to the HLA receptors, DQ2, and DQ8 (Schuppan 2000). The presentation of peptides to HLA-DQ2/DQ8 protein subunits on the surface of antigen-presenting cells (APC) in the presence of gluten-specific T cells induces both adaptive as well as an innate immune response in CD patients. The characteristics of CD are crypt hyperplasia, increased infiltration villous atrophy, increased lymphocyte infiltration and the stimulation of CD4+ T cells against gluten epitopes, and the production of tTG targeting autoantibodies (Dewar et al. 2004).

**Fig. 1:**
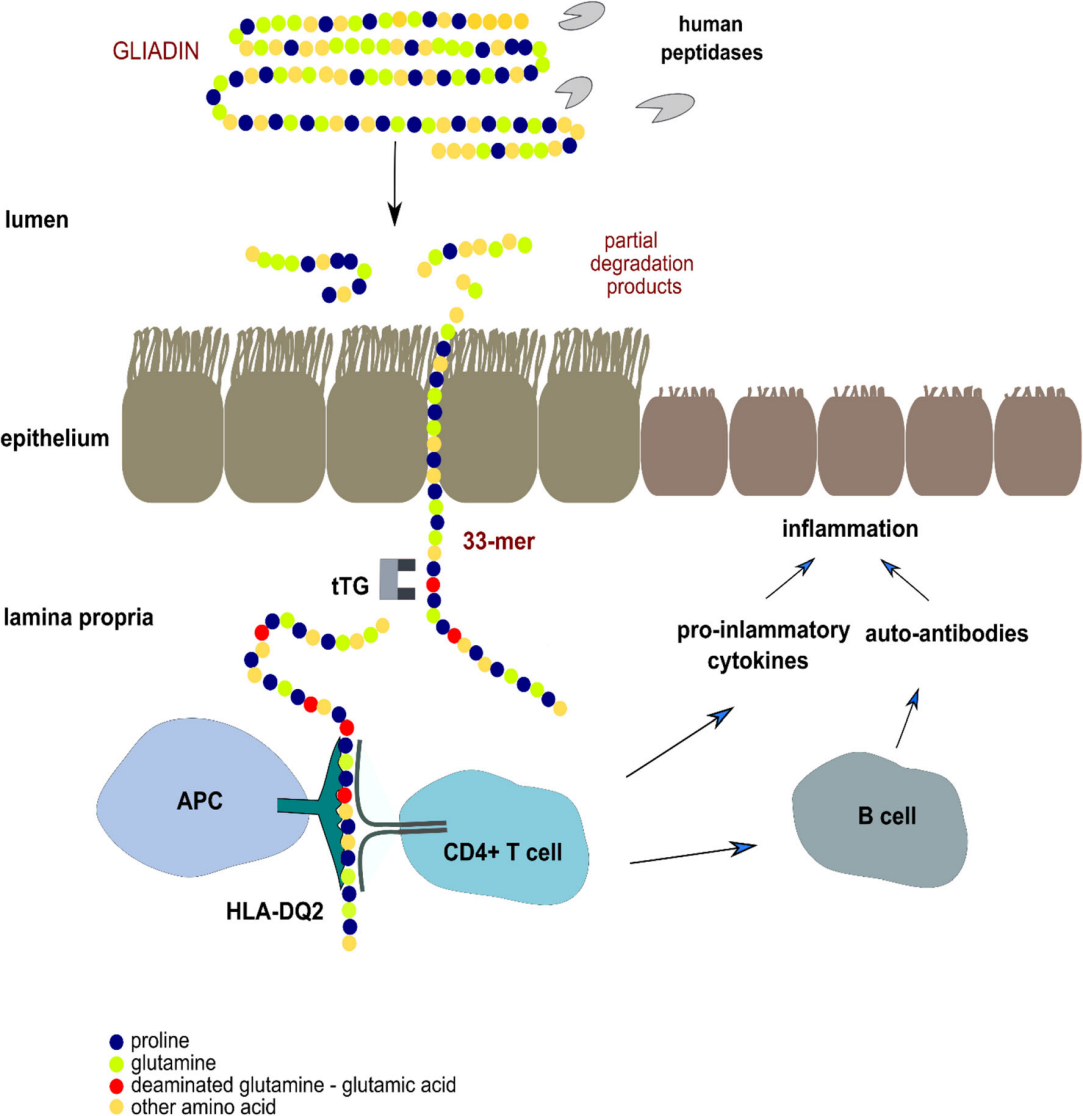
The resistance of gliadin to human proteolytic enzymes causes inflammation in human small intestine. Partially digested gliadin fragments cross the epithelium and reach the lamina propria. The most toxic fragment contains 33 amino acid residues mostly proline and glutamine. This peptide can bind to DQ2 molecules directly on the surface of antigen presenting cells (APC) (Qiao et al. 2005). Tissue transglutaminase (tTG) deaminates the glutamine residues to glutamic acid, thereby enhancing their affinity to the HLA receptor DQ2, that activates gliadin-specific T-cells. The result is chronic inflammation of the intestinal mucosa

**Fig. 2:**
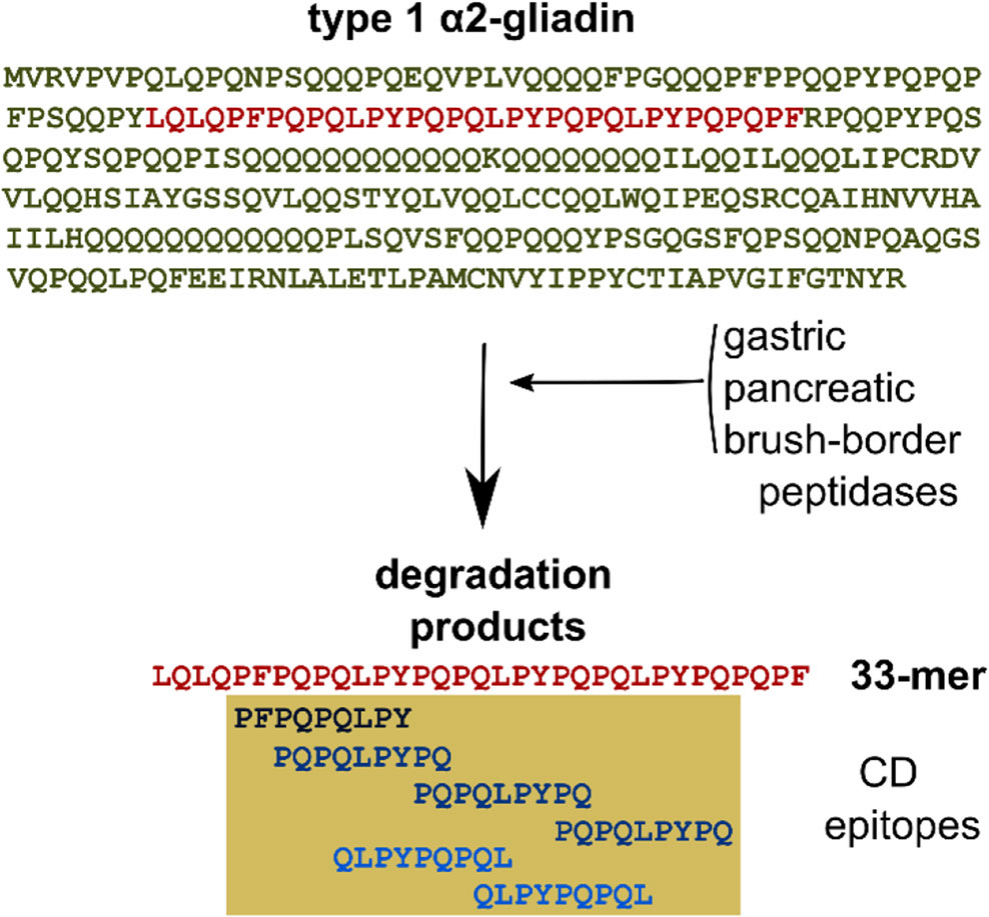
Proteolytic processing of the type 1 α2-gliadin. A 33-mer peptide fragment is resistant to the proteolytic enzymes in the human gastrointestinal tract. This peptide is the largest and most toxic degradation product. It contains six partially overlapping immunodominant CD epitopes that induce gluten-specific immune responses and intestinal inflammation

All gluten proteins (gliadin and glutenin from wheat, hordein from barley, secalin from rye, and avenin from oat) possess their own sets of toxic and immunogenic peptides (or epitopes) with distinct immunogenicity. However, gliadin peptides are known to be the most toxic and numerous, specifically derived from α- and γ-gliadin: the strongest and most common adaptive response to gluten is directed toward an α2-gliadin fragment of 33 amino acids in length containing six partially overlapping immunodominant CD epitope regions (Shan et al. 2002; Camarca et al. 2009) (Figs. 1 and 2). α-gliadins genes from diploid *Aegilops tauschii* (DD) introduced six types of α a-gliadins (named 1–6) into the currently used hexaploid wheat (AABBDD) (Ozuna et al. 2015; Schalk et al. 2017). Distinct types of α-gliadins differ mainly in the number of repeat blocks that contain the interspersed motifs PFPPQQ and PYPQPQ. Only type 1 α-gliadins contain the full immunodominant 33-mer epitopes.

### Wheat allergy

Wheat flour-triggered IgE-mediated food allergy is one of the top eight food allergies reported 0.2–1.0% globally (Cianferoni 2016). Wheat allergy (WA) is classified as a classic food allergy that is induced by wheat (not only gluten) intake and involves subtypes such as immediate wheat allergy, baker’s asthma, wheat contact dermatitis, and wheat-dependent exercise-induced anaphylaxis (Roszkowska et al. 2019). WA typically develops in childhood during early infancy and is frequently outgrown between the ages of 3 and 5 (Patel and Volcheck 2015).

IgE-mediated food allergies have been associated with sensitization to particular cereal storage prolamins or the non-gluten proteins within wheat, α-amylase inhibitors, thioredoxin, lipid transfer protein, profilin, and serpin, among others (Matsuo et al. 2015). A major allergenic protein from wheat is the ω5-gliadin repetitive region that consists almost entirely of the peptides FPQQQ and QQIPQQ (Battais et al. 2005; Denery-Papini et al. 2011). Other prolamins have been described to contain IgE binding epitopes including ω1, 2-gliadins, α-gliadins, γ- gliadins, LMW, and HMW subunits of glutenin (Denery-Papini et al. 2011; Battais et al. 2005).

### Non-celiac gluten sensitivity

Non-celiac gluten sensitivity (NCGS) may be defined as a gluten (wheat)-dependent disorder with symptoms similar to CD (abdominal pain, discomfort, bloating, altered bowel habit, and fatigue) but with mostly normal small intestinal histology and lack of anti-tTG autoantibodies. Prevalence of NGCS is highly variable 0.63–6.0% (although it is difficult to estimate because the condition lacks specific biomarker(s)) (Sapone et al. 2012). Its pathogenesis is still not clear; gluten triggers an innate immune response in NCGS and provokes an additional adaptive immune response with increased expression of IL-6, IL-21, IL-17, and IFN-γ. NCGS is more frequently diagnosed in adults rather than in children (Volta et al. 2014). Symptoms disappear after starting on a gluten-free diet and appear again after a gluten challenge within a few hours or a couple of days (Catassi et al. 2013; Barbaro et al. 2018).

## Peptidases able to degrade gluten

There is no cure for gluten intolerance disorders and the only treatment option is avoiding gluten in food, although even this does not eliminate all the symptoms. Providing enzymes able to degrade gluten and the immunogenic peptides is being investigated as a complementary treatment option. Peptidases can be used to eliminate gluten from food material (review Scherf et al. 2018). In addition, peptidases given as oral therapy have been tested to alleviate gluten-related conditions (Cerf-Bensussan et al. 2007; Tye-Din et al. 2010; König et al. 2017).

Enzymes that cleave the peptide bond are called peptidases or proteases. These enzyme names are often used as synonyms. Here, we call this class of enzymes peptidases. In the digestive tract, peptidases are responsible for cleavage of ingested proteins. Gluten-related health problems arise from the inefficiency of peptidases to degrade the immunogenic epitopes. Most peptidases, even those with low specificity, are unable to hydrolyze peptide bonds formed by proline residues due to the cyclic structure and the special constraints it exerts on the peptide backbone structure. The N-terminal amine of proline lacks a hydrogen, and the side chains are typically in the *cis* conformation. These properties impair the susceptibility of X↓P bonds (between any amino acid “X” and proline residue “P”) to proteolytic cleavage.

There are several peptidases from different classes that have been shown to degrade proline and gluten-rich prolamine molecules with varying efficiently. Peptidases can either act only near the ends of polypeptide chains (exopeptidases EC 3.4.11-19), or amidst polypeptide chains (endopeptidases EC 3.4.21-24 and EC 3.4.99). Here, we focus on endopeptidases that are more effective at gluten degradation than exopeptidases (Table 2). We use the classification of peptidases employed by MEROPS (http://merops.sanger.ac.uk.), a comprehensive database containing information on peptidase sequence, structure, substrates, and inhibitors (Rawlings et al. 2018).

**Table 2:** Most studied microbial endopeptidases cleaving proline- and glutamine-rich prolamine molecules

Enzyme family	Cleavage site	Substrate	Subcellular location	pH optimum	Temp. optimum (°C)	Microbe; peptidase	Origin	Reference
Metallopeptidase								
M4 elastase	Q↓L	Intact molecule	Extracellular	2.0–7.0	37	*Bacteria; Pseudomonas aeruginosa;* LasB	Human feces	(Wei et al. 2015; Caminero et al. 2016)
Serine peptidase								
S8 subtilisin	XPQ↓; LPY↓	Intact molecule	Extracellular	3–10	37	*Bacteria; Rothia spp.*	Human oral cavity	(Zamakhchari et al. 2011)
S9A prolyl-oligopeptidase	P↓F; P↓Y; P↓Q	Oligopeptide	Cytosolic	6.0–7.0	37	*Bacteria; Myxococcus xanthus*	Soil	(Shan et al. 2004; Kocadag Kocazorbaz and Zihnioglu 2017)
	P↓Q; P↓Y	Oligopeptide	Periplasmic	6.0–7.0	43	*Bacteria; Sphingomonas capsulata*	Distilled water	(Kabashima et al. 1998; Shan et al. 2004)
	GP↓; AP↓	Oligopeptide	Extracellular	6.0–8.0		*Bacteria; Chryseobacterium taeanense*	Rhizosphere of cereal crops	(de Amador et al. 2019)
	SAP↓; GGP↓; TP↓G; GP↓	ND	Cytosolic	6.0–9.0	37	*Bacteria; Cellulomonas sp.*	Human gut	(Kumar et al. 2018)
	P↓Q; P↓F; P↓Y	Oligopeptide	Cytosolic	5.0–8.0	63	*Bacteria; Sphaerobacter thermophilus*	Sewage sludge	(Pati et al. 2010; Shetty et al. 2017)
	GP↓; AP↓: AA↓	Intact molecule, oligopeptide	Not exported	∼7	85–90	*Archaea; Pyrococcus furiosus*		(Harwood et al. 1997; Harris et al. 2001)
S28 prolyl endopeptidase	XP↓	Intact molecule, oligopeptide	Extracellular	4.0–5.0	15–60	*Fungi; Aspergillus niger*		(Edens et al. 2005)
S53 serine-carboxyl peptidase	F↓P; Q↓L	Intact molecule	Extracellular	3.0–6.0	37	*Bacteria; Actinoallomurus* A8	Soil pH 4.2	(Cavaletti et al. 2019)

### Metallopeptidases

#### Family M4. Elastase (E.C.3.4.24.26)

Among metallopeptidases that degrade gluten, elastase LasB has gained significant attention because it is an extracellular metallopeptidase excreted from *Pseudomonas aeruginosa* that has been isolated from human feces (Wei et al. 2015) and the duodenum of CD patients (Caminero et al. 2016). *P. aeruginosa* is an opportunistic human pathogen that requires elastase to degrade mucins and surfactant proteins (Kuang et al. 2011) and exogenous flagellin which acts to prevent flagellin-mediated immune recognition (Casilag et al. 2015). Elastase LasB also efficiently hydrolyses gluten molecules, but produces a multitude of shorter immunogenic peptides that could activate gluten-specific T-cells in CD patients. It has been shown in germ-free mice that *Lactobacillus rhamnosus* and *Lactobacillus fermentum* isolated from the duodenum of non-CD humans degrade gluten peptides produced by *P. aeruginosa* peptidases thereby reducing their immunogenicity (Caminero et al. 2016). The peptidase activity was provided by the exopeptidases (of other peptidase families, not metallopeptidases) that *Lactobacillus* spp. often produce.

### Serine peptidases

Most of the gluten-degrading bacterial peptidases described so far belong to the serine family of proteases.

#### **Family S8**. Subtilisin

Peptidase family S8 contains the serine endopeptidase subtilisin and its homologs that cleave after the XPX↓ motif. Most of these enzymes are inefficient against many immunogenic gluten sequences.

However, the enzyme from *Rothia* spp. is active against the 33-mer immunogenic peptide. *Rothia aeria* is a commensal bacterium that inhabits the human oral cavity and is active over much of the intestinal pH range (pH 3–10) (Zamakhchari et al. 2011). *Rothia* subtilisins are unique in that they cleave both XPQ↓ and LPY↓.

Two S8 subtilisins from *Bacillus licheniformis*, subtilisin A (sold by Sigma) and the food-grade Nattokinase (extracted from a dietary food supplement), degrade the immunogenic gliadin-derived 33-mer peptide and the immunodominant epitopes recognized by the R5 (QQPFP and related sequences) and G12 (QPQLPY) antibodies. Nattokinase abolishes the R5 epitopes but is less effective in eliminating the G12 epitopes (Wei et al. 2016).

#### **Family S9, Serine-carboxyl peptidase (sedolisin, E.C.3.4.21.100)**

Soil *Actinomycete Actinoallomurus* A8 produces extracellular sedolisin that has optimal pH around 3. This enzyme cleaves both F↓P and Q↓L in the 33-mer as well as intact gliadin proteins. Its recombinant active form has been produced in *Streptomyces lividans* TK24, a strain generally regarded as a safe (GRAS) and a source of proteins for human alimentary use (Cavaletti et al. 2019).

Many natural sedolisins are not very efficient in degrading gluten; however, it has been demonstrated that their properties can be improved by engineering. Kumamolisin from *Alicyclobacillus sendaiensis*, with an optimal pH around 3, is a serine-carboxyl peptidase (S53) with collagenase activity (Tsuruoka et al. 2003). After molecular engineering of kumamolisin to shift its cleavage specificity from P↓R or P↓K to P↓Q, a mutant termed Kuma030 with high proteolytic activity against gluten was obtained (Gordon et al. 2012).

#### Family S9, subfamily S9A, prolyl oligopeptidase (POP, E.C.3.4.21.26)

These enzymes are specific for cleavage after proline residues, thus making this group of peptidases very synergistic with human digestive proteases. The S9A subfamily of enzymes are also referred to as prolyl endopeptidases (PEP). These enzymes have been extensively studied and are already being developed for medicinal claims. POP from the bacterium *Sphingomonas capsulata* (SC-PEP) (cleaves after P) has been combined with the barley cysteine endopeptidase PEP-B2 (cleaves after glutamine) to form a combined enzymatic activity that attacks the immunogenic peptides after the most frequent amino acid residues (P and Q). This formulation, developed under the name Latiglutanase, is expected to provide synergy and create a more active medication (Lähdeaho et al. 2014).

Generally, POP hydrolyses oligopeptides that consist of less than 30 residues (Camargo et al. 1997). However, POP from the thermophilic archaeon *Pyrococcus furiosus* has been shown to cleave larger substrates (Harwood et al. 1997; Harris et al. 2001). The *Sphaerobacter thermophilus* enzyme also seems to cleave full intact gluten molecules, although the enzymatic assay was performed in a complex mixture that also contains malt (Shetty et al. 2017). The POP studied so far originate from a wide range of environmental bacteria and archaea from phyla/class: *Actinobacteria* (*Cellulomonas* sp.), *Bacteroidetes* (*Flavobacterium meningosepticum*, *Chryseobacterium taeanense*), *Alphaproteobacteria* (*Sphingomonas capsulata*), *Deltaproteobacteria* (*Myxococcus xanthus*), *Chloroflexi* (*Sphaerobacter thermophilus*), and *Euryarchaeota* (*Pyrococcus furiosus*) (Table 2). All these enzymes can detoxify the 33-mer peptide but differ in their hydrolytic activity and stability under in vitro artificial gastrointestinal conditions (acidic pH, pancreatic proteases, and membrane peptidases of the small intestinal mucosa).

Based on a bioinformatic analysis, Kaushik and Sowdhamini (2014) showed that POP are widely distributed in all classes of bacteria and archaea with diverse domain architectures. Several of the proteins contain signaling peptides for export, which suggests their periplasmic or extracellular location (Table 2).

Unfortunately, the strains that produce promising POP enzymes cannot be used in the food industry because of their environmental origin. Lactic acid bacteria are food-grade and have beneficial gut properties and resistance to harsh gut conditions. Recombinant *Lactobacillus casei* strains were constructed to deliver POP from *M. xanthus* to the gut. It was found that the strain that secretes POP into the extracellular medium more effectively degrades the 33-mer oligopeptide compared to the strain that retained POP to the intracellular environment. The probable reason is that the extracellular enzymes can directly attack the peptide and do not require uptake events prior to cleavage (Alvarez-Sieiro et al. 2014). Another group used *Lactococcus lactis* as delivery vector for secreting *F. meningosepticum* POP into the growth medium (Lim et al. 2017).

### Other enzymes of nonhuman origin

It is expected that gluten degradation occurs in environments where gluten occurs naturally, i.e., in cereal plant grains and in organisms who use it as a food source. Endogenous cereal proteases that hydrolyze storage proteins, including gluten, are synthesized during germination. The well-studied cysteine endopeptidase B2 of barley cuts at glutamine residues and prefers intact proteins as a substrate (Bethune et al. 2006; Osorio et al. 2019). A combination of the barley cysteine endopeptidase EP-B2 with the bacterial POP from *F. meningosepticum-*PEP has been proven to be effective in detoxifying gluten proteins. Site saturation mutagenesis effectively increased the thermostability of *F. meningosepticum*-PEP, which should allow it to maintain its enzymatic activity in the core of bread during baking, where temperatures generally do not exceed 100°C (Osorio et al. 2020).

Gluten-degrading peptidases are also found in organisms involved in the decomposition of organic matter. Family S28 prolyl endopeptidases from the fungus *Aspergillus niger* and edible mushroom in the family *Physalacriaceae*, *Flammulina velutipes*, are unique in the serine peptidase family because they have endopeptidase activity that cleaves after XP (Edens et al. 2005; Stepniak et al. 2006; Kang et al. 2013; Schulz et al. 2018). *Aspergillus niger* AN-PEP is active between a pH of 2 and 8, with an optimal activity between pH 4 and 5. It is not degraded by pepsin, and thereby remains functional in the stomach (Stepniak et al. 2006; Mitea et al. 2008). It has been shown that AN-PEP significantly degrades most gluten in the stomach before it enters the duodenum (Salden et al. 2015). Capsules containing the AN-PEP enzyme have been marketed for over a decade, although the health effects have been disputed (Krishnareddy et al. 2017).

For *Tenebrio molitor*, *Rhizopertha dominica*, and related cereal-feeding insects, the main dietary proteins are the storage proteins of cereal grains. Post-glutamine cleaving peptidases were isolated from the midgut of the pest, *Tenebrio molitor* (yellow mealworm) (Goptar et al. 2013). POP from the gastrointestinal system of the beetle *R. dominica* cleaves proline-rich peptides from wheat and barley in P↓Q and P↓Y (Mika et al. 2015).

## Gluten-degrading bacteria in human digestive tract

There are thousands of bacterial species that inhabit the human digestive tract. Several of these bacteria can potentially degrade gluten and a healthy microbiome composition could modulate the symptoms of gluten-related diseases.

It ought to be noted that the microbiome composition is mostly investigated by the 16S rRNA gene marker. Because different strains of the same bacterial species can have very different proteolytic activities, 16S-based studies must be complemented with cultivation and metagenomics-based approaches to assess proteolytic activity. In addition, the 16S rRNA-based approaches have shown that the bacterial communities are very different in different parts of the digestive tract. Here, we discuss the gluten-degrading potential of these different communities.

### Oral cavity

The digestion of food starts in the oral cavity and it has been shown that bacteria from the oral cavity have the capacity to degrade gluten. There are approximately 800 prokaryotic species in the oral cavity, of which 70% were found to be cultivable (Verma et al. 2018). *Rothia* spp., *Actinomyces odontolyticus*, *Neisseria mucosa*, and *Capnocytophaga sputigena* were found to have the highest activity against gliadin or the 33-mer immunogenic peptide (Fernandez-Feo et al. 2013). *Rothia* spp. enzymes have been studied in more detail (see above). Although their activities are quite low, the high abundance of *Streptococcus* sp. could still substantially contribute to the overall gliadin-degrading capacity in the human mouth (Aas et al. 2005; Fernandez-Feo et al. 2013). The bacterial strains studied to date have considerably different peptidase activities, which suggests that efficient gluten degradation can be achieved in combination. Approximately, 1 l of saliva, which contains a diverse collection of aerobic and anaerobic bacteria, is produced on a daily basis (Maukonen et al. 2008). Saliva contains a high amount of proline-rich proteins; the degradation of which most likely starts by the subtilisins produced by oral bacteria (Messana et al. 2008; Helmerhorst et al. 2008). Therefore, it is probable that some of these bacteria can also contribute to the degradation of gluten. Saliva is swallowed and thereby also provides substrates for bacteria in the lower digestive tract.

### Stomach

The pH of the stomach after a meal is in the range of 2–4. This vastly restricts the bacterial groups that can be active in the stomach or even pass its harsh conditions alive. In stomach, the microbial density is ca 10^2^ to 10^4^ cfu/ml. The gastric microbiota is diverse and includes species from the genera *Streptococcu*s, *Propionibacterium*, *Lactobacillus*, *Staphylococcus*, *Prevotella*, *Veillonella*, *Rothia*, and *Haemophilus* (Delgado et al. 2013; Nardone and Compare 2015). Although there are no studies on the capacity of stomach bacteria to degrade gluten, it has to be noted that *Rothia* spp. can have activity at low pH and therefore could be involved in gluten degradation in the stomach.

### Small intestine

The degradation of nutrients continues in the duodenum, jejunum, and ileum of the small intestine. Many degradation products are absorbed in the small intestine, which also performs strong immunomodulatory functions. In line with this, CD is triggered in the small intestine. The conditions in the duodenum can also be harsh for certain bacteria because it contains high levels of human proteases and bile acids. This is probably the reason why the bacterial count is lower in duodenum (10^1^–10^5^ CFU/ml) and rises in the jejunum (10^4^–10^7^ CFU/ml) and ileum (10^3^–10^8^ CFU/ml) (O’Hara and Shanahan 2006; Leser and Mølbak 2009; Zoetendal et al. 2012). The physiological bacterial communities within the small intestine are difficult to study because of the need to take biopsy is needed. Therefore, only one study has been published to date that assesses the gluten degradation potential of bacterial communities in small intestine. This work is restricted to the duodenum.

Bacteria from biopsy samples from the duodenum were investigated for their activity when degrading intact gluten and the 33-mer peptide (Herrán et al. 2017). This study showed that 15 bacterial species from genera *Actinomyces*, *Bacillus*, *Lactobacillus*, *Prevotella*, *Pseudomonas*, and *Stenotrophomonas* were able to hydrolyze the 33-mer. Additionally, bacteria that showed extracellular gluten-degrading activity were as follows: *Bacillus licheniformis*, *B. subtilis*, *B. subtilis/amyloliquefaciens*, *Bacillus pumilus*, *L. casei/paracasei*, *Pseudomonas aeruginosa*, *S. aureus*, *S. epidermidis*, *Stenotrophomonas maltophilia*, *Streptococcus salivarius/thermophilus*, and *Virgibacillus pantothenticus*. Overall, 60% of the bacteria identified were *Lactobacilli*. Because of their auxotrophy for numerous amino acids, *Lactobacilli* hydrolyze proteins through the action of their proteolytic system in order to get the amino acids required for their growth. *Lactobacilli* mostly produce cell wall attached exopeptidases that are relatively inefficient in eliminating gluten and gluten-derived peptides (Scherf et al. 2016). Still, *L. helveticus* has serine endopeptidases that belong to the subtilisin family that break down larger proteins into oligopeptides that are transported into the cytoplasm (Griffiths and Tellez 2013). Subsequently, the internalized peptides, 2 to 9 amino acid residues, are degraded into amino acids by the combined action of numerous internal peptidases that vary depending on the species (Savijoki et al. 2006; Raveschot et al. 2018). No single *Lactobacillus* strain possesses all the peptidases required to degrade toxic gluten oligomers. Gluten, and its shorter immunogenic peptides, can be degraded by a combination of bacteria with complementary peptidase activities (Francavilla et al. 2017).

### Colon

Colonic bacteria are characterized by studying feces. The estimated bacterial count in feces is ca 10^10^–10^12^ CFU/ml, and the number of cultivable species is estimated to be over 1500 (O’Hara and Shanahan 2006; Lagier et al. 2016).

Early work with human gut contents by Macfarlane identified fecal bacteria with proteolytic activity: *Propionibacterium*, *Clostridium*, *Streptococcus*, *Bacillus*, and *Staphylococcus*. Extracellular proteases were formed by *Streptococcus faecalis* ST6, *Propionibacterium acnes* P6, *Clostridium perfringens* C16, *C. bifermentans* C21, and *C. sporogenes* C25 (Macfarlane and Allison 1986; Macfarlane et al. 1988; Gibson et al. 1989).

In 2014, Caminero et al. (2014) identified gluten-degrading activity in strains from *Bacillus licheniformis*, *B. subtilis*, *B. pumilus*, *Bifidobacterium longum*, *Clostridium sordellii*, *C. perfringens*, *C. botulinum/sporogenes*, *C. butyricum/beijerinckii*, *Enterococcus faecalis*, *E. faecium*, *Propionibacterium acnes*, *Pediococcus acidilactici*, *Paenibacillus jamilae*, *Staphylococcus epidermidis*, *S. hominis*, *and Stenotrophomonas maltophilia*.

The importance of the prolamin degrading activity in colon is questionable because the damaging activity of gluten-derived epitopes already occurs in the duodenum.

## Probiotics in CD

Currently, there is no clear understanding of the role of the gut microbiota in CD. Several studies have reported changes in the composition of gut microbiota, “intestinal dysbiosis,”,in CD patients compared to healthy subjects (Nadal et al. 2007; de Palma et al. 2012; Cheng et al. 2013). A balanced composition of the human commensal microbiota has been considered crucial for the development of a healthy immune system; however, it is not clear if intestinal dysbiosis is the cause or effect of CD (Chibbar and Dieleman 2019). Probiotics have been considered as a strategy to modulate the gut microbiome in CD patients. Probiotics can influence gluten intolerance by digesting the gluten proteins to non-immunogenic small polypeptides, maintain the intestinal barrier by preventing immunogenic polypeptides from accessing the mucosa, and by regulating the immune system. The potential of several probiotics has been investigated. De Angelis and others reported the potential benefits of a probiotic cocktail with eight strains (VSL#3), *Bifidobacterium breve*, *Bifidobacterium infantis*, *acidophilus*, *Lactobacillus plantarum*, *Lactobacillus casei*, *Lactobacillus delbrueckii* subsp. *bulgaricus*, *Streptococcus thermophilus*, and *Bifidobacterium longum*, which decreased wheat-induced discomfort (De Angelis et al. 2006). It ought to be noted that the probiotic cocktail was added during the food-processing step and could produce predigested and thus tolerable gliadins that increase the palatability of gluten-free products. Importantly, as mentioned above, individual *Lactobacillus* strains are not capable of complete detoxification of gluten-derived peptides (Francavilla et al. 2017). The same is true for VSL#3; individual probiotic strains were inadequate to break down gliadin compared to the efficiency with a pool of strains (De Angelis et al. 2006; Harnett et al. 2016).

*Bifidobacterium* spp. has been shown to modulate a proinflammatory milieu of CD in several in vitro studies (Medina et al. 2008; Lindfors et al. 2008; Laparra et al. 2012). *Bifidobacterium* spp. also helped to restore the healthy percentage of the main microbial components (Quagliariello et al. 2016). It would be prudent to study the effects of *Bifidobacterium* spp. on CD in further clinical studies.

It is expected that probiotics will not provide a rapid cure for complex diseases such as CD, but rather alleviate the severity and symptoms. More studies are needed that specifically address how the gut microbiome can modulate or alter the course of the disease. We expect that probiotics will probably need to be combined with long-term dietary changes.

## Natural sources of gluten-degrading bacteria

CD is an autoimmune disease triggered by gluten and most probably other environmental factors not yet defined. CD prevalence has increased over time in geographical regions characterized by a Western lifestyle (Catassi 2015). In Western society, the gut microbiome has changed when compared to the traditional societies. The “Biodiversity hypothesis” states that interaction with diverse and abundant environmental microbiota is important for the prevention of immune-mediated non-communicable diseases (von Hertzen et al. 2011; Hanski et al. 2012). There are a rising number of studies that show that a living environment, and changes therein, likely shape the skin as well as the composition of gut microbiota and are important for the development of a normal immune system and protection from immune-mediated diseases (Seiskari et al. 2007; Stein et al. 2016; Lehtimäki et al. 2017; Hui et al. 2019). For example, diverse vegetation around homes, particularly shrubs and non-woody flowering plants, and the coverage of built areas are associated with health-related changes in gut microbiota composition (Parajuli et al. 2020). The effect of environmental bacteria on allergies has been associated with their immunomodulatory effects (Nurminen et al. 2018; Ottman et al. 2019; Roslund et al. 2020).

Empirical studies have implicated bacteria as the dominant contributor to proteolytic activity in soils (Watanabe and Hayano 1993; Nguyen et al. 2019). Bacterial isolates from *Bacillus*, *Pseudomonas*, and *Flavobacterium-Cytophaga* have been shown to be important agents of proteolysis and act as the main sources of soil peptidase activity (Bach and Munch 2000; Vranova et al. 2013). Given the nature of nitrogen limitation in most soils and aquatic environments, the ability to readily break down high molecular weight proteinaceous material into amino acid precursors for cell growth or energy generation would be highly favorable (Geisseler et al. 2010; Kolton et al. 2013).

Root vegetables are in direct contact with soil and share some of the surrounding microbiome. We have recently shown in an in vitro study that bacteria that originate from root vegetables can degrade gluten and detoxify the toxic immunogenic CD epitopes at 37°C under microoxic conditions (Kõiv et al. 2020). Bacteria with strong extracellular protease activity were identified as *Bacillus pumilus*, *Bacillus cereus*, *Bacillus subtilis*, *Bacillus circulans*, *Bacillus licheniformis*, *Bacillus psychrosaccharophilus*, *Clostridium bifermentans*, *Clostridium sporogenes*, and *Clostridium subterminale*. Four bacterial strains belonging to the species *Bacillus pumilus*, *Clostridium subterminale*, and *Clostridium sporogenes* produce peptidases with post-proline cleaving activity that successfully neutralized the toxic immunogenic epitopes. The bacterial groups identified as 33-mer and gluten degrades are similar or even identical to the bacteria degrading these substrates in duodenum and feces (described above in the “Gluten-degrading bacteria in human digestive tract” section). Therefore, it is possible that these strains are introduced to the gastrointestinal tract together with food. Currently, it is not possible to estimate if these bacteria inhabit the gastrointestinal tract stably, perhaps with specific required dietary substrates, or are constantly introduced with food.

It is not known if the observed peptidase activity is provided by one enzyme or a mixture of several enzymes. As mentioned above, *B. pumilus* and *C. sporogenes* isolated from duodenum and feces hydrolyze both the 33-mer, and have also proteolytic activity against intact gluten proteins (Macfarlane et al. 1988; Caminero et al. 2014; Herrán et al. 2017). Both bacteria produce several peptidases that degrade other proline and glutamine-rich proteins such as keratin (7.5% proline and 12% glutamine) (Ionata et al. 2008; Fellahi et al. 2016). The complete degradation of keratin to single amino acids requires the synergistic action of (at least) three kinds of peptidases, namely endo-, exo-, and oligo-peptidase (Qiu et al. 2020). It is probable that the natural degradation of gluten also occurs by a combination of different enzymes although this hypothesis requires further investigation. It is also probable that gluten degradation activity can be achieved by enzymes that usually degrade certain proline-rich proteins such as keratin, collagen, and elastin. It is described above that elastase from *P. aeruginosa* can degrade gluten. The gluten-degrading activity of human gastrointestinal elastases has also been described (Gutiérrez et al. 2017).

The human digestive tract is saturated with easily accessible sources of nitrogen for bacteria. Therefore, only a few microbes that colonize the digestive tract “waste” energy on the production of extracellular proteases. Environmental bacteria are more active in using extracellular proteases and, when introduced into the gut, could provide beneficial proteolytic activity.

In addition, opportunistic pathogens sometimes produce high levels of peptidases that could also degrade gluten (*P. aeruginosa*, *Stenotrophomonas maltophilia*, *C. perfringens*, *Enterococcus faecalis*, *E. faecium*) (Caminero et al. 2014; Caminero et al. 2016). CD is largely a contemporary disease. It has been noted that oral microbiomes from hunter-gatherers and traditional farmers reveal shifts in commensal balance and pathogen load linked to diet: species found preferentially in hunter-gatherers included microbes often considered as oral pathogens, despite their hosts’ apparent good oral health (Lassalle et al. 2018). It is possible that also in contemporary diets, microbes considered as opportunistic pathogens can contribute to degradation of gluten-derived peptides.

In conclusion, the enzymatic food supplements and probiotics are being developed to alleviate the symptoms of gluten intolerance. In addition, natural bacterial communities from food could also facilitate detoxification of gluten. Complete gluten degradation is a complex process that can be achieved with assistance of various combinations of enzymes produced by a wide range of microorganism, including bacteria. These bacterial communities could facilitate adaptation to our consumption of crop products that were introduced rather recently to humankind.
